# C-Fiber Degeneration Enhances Alveolar Macrophage-Mediated IFN-α/β Response to Respiratory Syncytial Virus

**DOI:** 10.1128/spectrum.02410-22

**Published:** 2022-11-09

**Authors:** Yu He, Changgen Li, Zhili Wang, Zhongying Yang, Jianhua Wei, Luo Ren, Yu Deng, Shiyi Chen, Zhixu Ye, Na Zang, Enmei Liu

**Affiliations:** a Department of Respiratory Medicine, Children’s Hospital of Chongqing Medical University, Chongqing, China; b National Clinical Research Center for Child Health and Disorders, Chongqing, China; c Ministry of Education Key Laboratory of Child Development and Disorders, Chongqing, China; d Chongqing Key Laboratory of Pediatrics, Chongqing, China; e Department of Central Laboratory, Guizhou Provincial People’s Hospital, Guizhou Province, China; University of Manitoba

**Keywords:** alveolar macrophages, C fibers, respiratory syncytial virus, transient receptor potential vanilloid 1, vasoactive intestinal peptide

## Abstract

Stimulation of unmyelinated C fibers, the nociceptive sensory nerves, by noxious stimuli is able to initiate host responses. Host defensive responses against respiratory syncytial virus (RSV) infection rely on the induction of a robust alpha/beta interferon (IFN-α/β) response, which acts to restrict viral production and promote antiviral immune responses. Alveolar macrophages (AMs) are the major source of IFN-α/β upon RSV infection. Here, we found that C fibers are involved in host defense against RSV infection. Compared to the control mice post-RSV infection, degeneration and inhibition of C fibers by blockade of transient receptor potential vanilloid 1 (TRPV1) lowered viral replication and alleviated lung inflammation. Importantly, AMs were markedly elevated in C-fiber-degenerated (KCF) mice post-RSV infection, which was associated with higher IFN-α/β secretion as measured in bronchoalveolar lavage fluid (BALF) samples. Degeneration of C fibers contributed to the production of vasoactive intestinal peptide (VIP), which modulated AM and IFN-α/β levels to protect against RSV infection. Collectively, these findings revealed the key role of C fibers in regulating AM and IFN-α/β responses against RSV infection via VIP, opening the possibility for new therapeutic strategies against RSV.

**IMPORTANCE** Despite continuous advances in medicine, safe and effective drugs against RSV infection remain elusive. As such, host-RSV interactions and host-directed therapies require further research. Unmyelinated C fibers, the nociceptive sensory nerves, play an important role in regulating the host response to virus. In the present study, from the perspective of neuroimmune interactions, we clarified that C-fiber degeneration enhanced the AM-mediated IFN-α/β response against RSV via VIP, providing potential therapeutic targets for the treatment of RSV infection.

## INTRODUCTION

Human respiratory syncytial virus (RSV) is an enveloped, single-stranded, negative-sense RNA virus of the *Orthopneumovirus* genus in the *Pneumoviridae* family (1). RSV infection is a common cause of bronchiolitis and a major/significant contributor to infant deaths under the age of 1 year (2) and of morbidity and mortality in elderly and immunocompromised people (3). Despite continuous advances in medicine, safe and effective drugs against RSV infection remain elusive, largely due to viral escape from therapies following genetic changes and mutations (4). As such, host-RSV interactions and host-directed therapies require further research.

Alpha/beta interferon (IFN-α/β) are important protective factors during infection, and robust host IFN-α/β responses are associated with milder RSV infection in infants (5). IFN-α/β induce IFN-stimulated genes to mediate antiviral effects and initiate the early production of proinflammatory cytokines to protect against RSV infection (6). However, RSV employs multiple strategies to hamper IFN-α/β expression by virtue of its nonstructural 1 (NS1) and NS2 proteins (7). Alveolar macrophages (AMs) are the main source of IFN-α/β production following RSV infection (8). Deleting AMs significantly reduces IFN-β production and promotes viral replication following RSV infection in mice (9). Thus, promoting endogenous IFN-α/β secretion from AMs in hosts could be of great therapeutic potential against RSV infection.

The airways and lungs are dominantly innervated by unmyelinated C-fiber sensory nerves primarily located under respiratory epithelial cells. C fibers express key receptors (e.g., TRPV1) that can respond to a variety of endogenous and exogenous agents, such as inhaled cigarette smoke, immune cell-derived factors, hydrogen ions, adenosine, reactive oxygen species (ROS), capsaicin, changes in osmolarity and temperature, etc. (10–12). Once activated, C fibers regulate the secretion of neuropeptides with different functions. Proinflammatory neuropeptides, such as substance P (SP) and calcitonin gene-related peptide (CGRP), are involved in neurogenic inflammation (13, 14). On the other hand, anti-inflammatory neuropeptides, such as vasoactive intestinal peptide (VIP), exert potent anti-inflammatory and bronchodilatory effects and play a role in maintaining pulmonary homeostasis (15, 16). Among these peptides, VIP was reported to suppress human immunodeficiency virus (HIV) replication in macrophages (17, 18) and counteract HIV-induced neuronal death (19, 20). In addition, VIP can also inhibit severe acute respiratory syndrome coronavirus 2 (SARS-CoV-2) RNA synthesis/replication in human monocytes and viral production in lung epithelial cells (21). These studies suggest that VIP may participate in host immune response against virus infection. Transient receptor potential vanilloid 1 (TRPV1) is a nonselective transient receptor potential channel that is highly expressed in sensory nerves (22). RSV is reported to upregulate TRPV1 expression in sensory neurons *in vitro* (23). Activation of TRPV1 expression in sensory neurons *in vitro* promotes herpes simplex virus replication (24). Besides this, TRPV1 may also be involved in airway hyperresponsiveness and hypersecretion in asthmatic mice post-RSV infection (25). Therefore, we speculate that C fibers may play an important role in regulating the host response post-RSV infection.

We previously demonstrated that C-fiber degeneration reduces viral titers and alleviates the inflammatory response upon RSV infection in mice (26, 27). In the present study, we aimed to further clarify the effects and mechanism of C fibers in host anti-RSV responses, providing a novel perspective on neuroimmune interactions and highlighting the functional role of the nervous system in the host defense against viral infection.

## RESULTS

### C fibers impair host protection against RSV infection.

**FIG 1:**
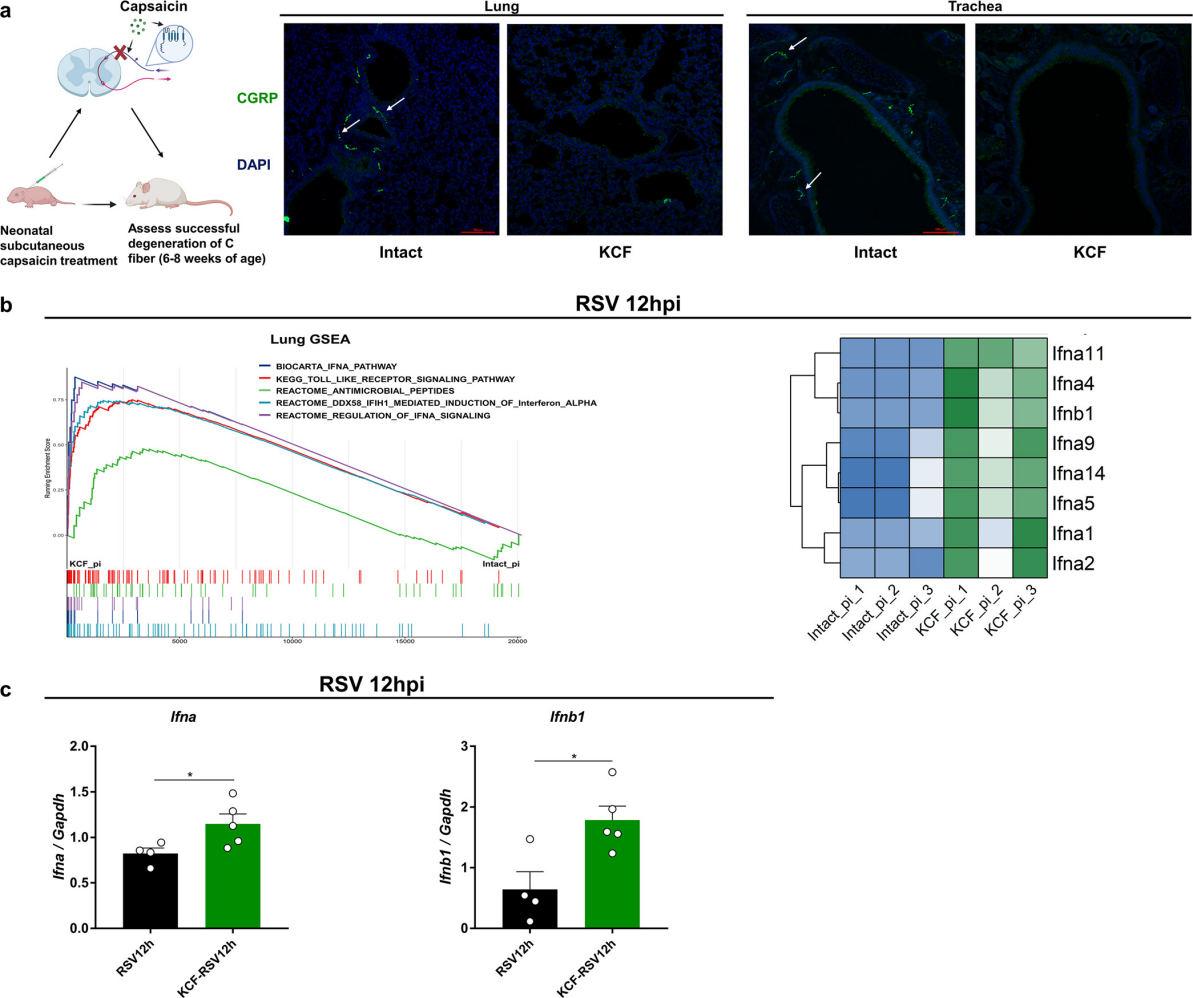
C fibers impair host protection against RSV infection. (a) Representative images of immunofluorescence staining of CGRP^+^ C fibers (green) in lungs of 6- to 8-week-old KCF and intact mice. White arrows points to CGRP^+^ C fibers. Nuclei (blue) were stained with the DNA dye DAPI (4′,6-diamidino-2-phenylindole). Scale bar = 100 μm. (b) Lung-enriched pathways based on GSEA of KCF and intact mice at 12 h post-RSV infection (RSV 12hpi) using RNA-seq data (left). Heatmap of *Ifna* and *Ifnb* based on RNA-seq (right). (c) *Ifna* and *Ifnb* mRNA expression levels based on qRT-PCR. pi, postinfection. *, *P < *0.05, and **, *P *< 0.01, compared with RSV group. Data are representative of 3 independent experiments. Error bars show standard errors of the means (SEM).

To study the role of C fibers in host defense against RSV infection, we established a C-fiber knockout (KCF) mouse model by administering capsaicin to newborn mice subcutaneously (s.c.). CGRP is a peptide commonly used as a marker of C fibers (28, 29). Here, CGRP-positive (CGRP^+^) C fibers were mostly lost in the lung and trachea of KCF mice compared with the number in mice with intact C fibers (intact mice) (Fig. 1a). Our previous study showed lower viral titers and lung inflammation in KCF mice (26, 27). Here, we conducted RNA sequencing (RNA-seq) of lung samples from KCF and intact mice at 12 h post-RSV infection. Gene set enrichment analysis (GSEA) results showed that pathways related to IFN-α/β and antimicrobial peptides were significantly enriched in the KCF post infection group compared with the levels in the intact post infection group (Fig. 1b). Furthermore, IFN-α/β mRNA expression was elevated in KCF mice, based on transcriptional profiling and real-time quantitative PCR (qRT-PCR) of IFN-α/β-encoding genes *Ifna* and *Ifnb1* (Fig. 1c). These results, combined with those of our previous study, suggest that C fibers may regulate the host antiviral response post-RSV infection.

**FIG 2:**
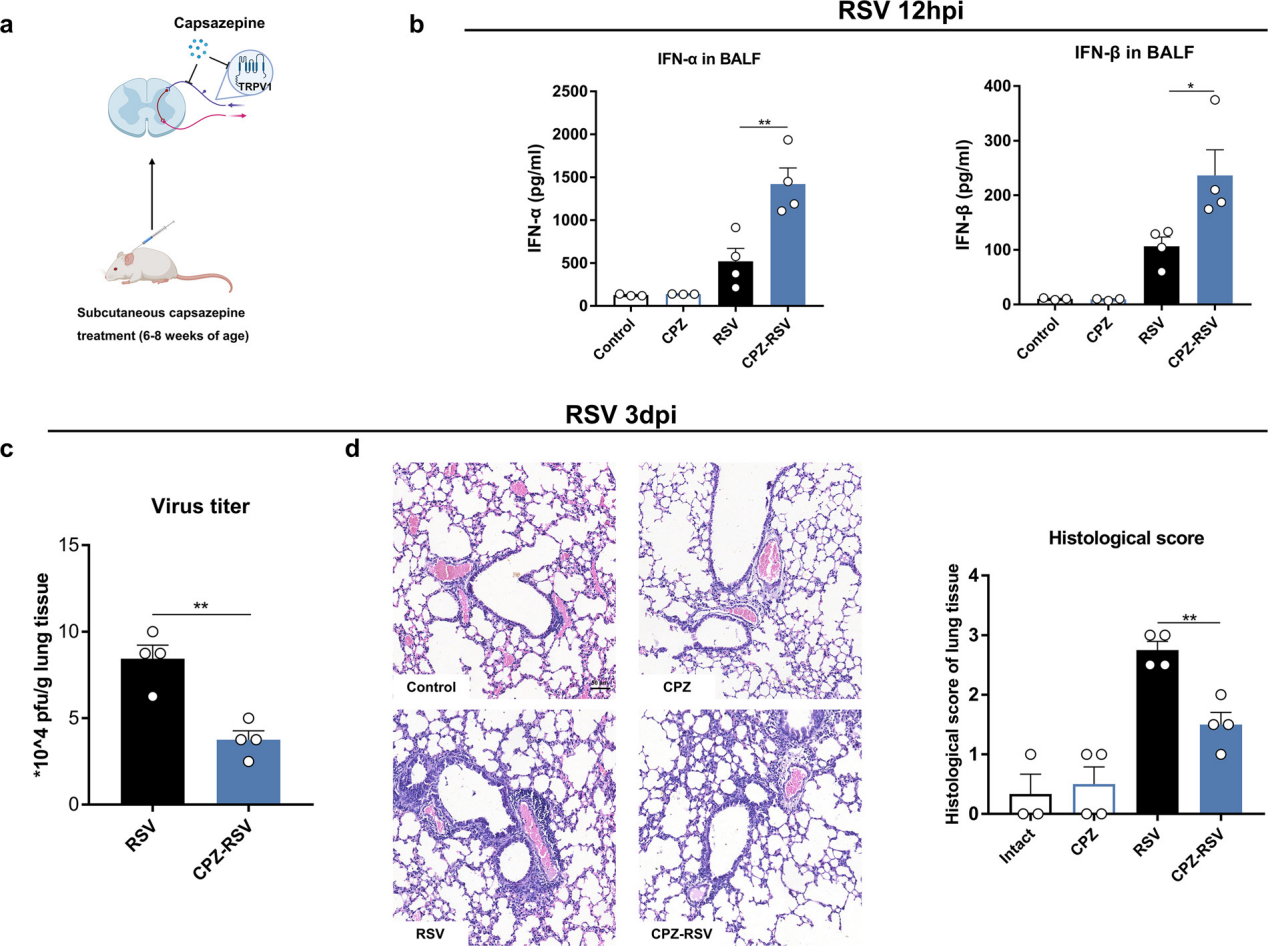
C fibers impair host protection against RSV infection. (a) Treatment with TRPV1 inhibitor CPZ in intact mice. (b) IFN-α/β secretion in BALF with or without CPZ treatment. (c) RSV titers in lung tissue with or without CPZ treatment. pfu, plaque-forming units. (d) Representative lung histopathological images and scores with or without CPZ treatment. Scale bar = 50 μm. *, *P < *0.05, and **, *P *< 0.01, compared with RSV group. Data are representative of 3 independent experiments. Error bars show SEM.

We next used complementary and nondevelopmental strategies to target C fibers by subcutaneous injections of capsazepine (CPZ), a potent TRPV1 antagonist (Fig. 2a). CPZ treatment promoted IFN-α/β production as measured in BALF (Fig. 2b) and reduced RSV titers (Fig. 2c). CPZ treatment also significantly decreased the RSV N gene mRNA and protein expression levels (Fig. S1a and b in the supplemental material). Lung inflammation was significantly alleviated by CPZ treatment, as indicated by alveolar wall thickness, peritracheal and blood vessel inflammatory infiltration, and histopathological score (Fig. 2d).

In contrast, subcutaneous injections of low-dose capsaicin (CAP), a potent TRPV1 agonist, inhibited IFN-α/β production as measured in BALF and promoted RSV replication and lung inflammation (Fig. S2a to d). Therefore, using two different targeting approaches, we found that C fibers impair host protection against RSV infection.

### C-fiber degeneration regulates AMs to mediate resistance against RSV infection.

As the main resident immune cells in the alveolar and interstitial spaces of the lung, AMs are the first line of host defense against respiratory pathogen invasion in the airways (30). AMs are also a major source of IFN-α production after RNA virus infection of the lung (31).

**FIG 3:**
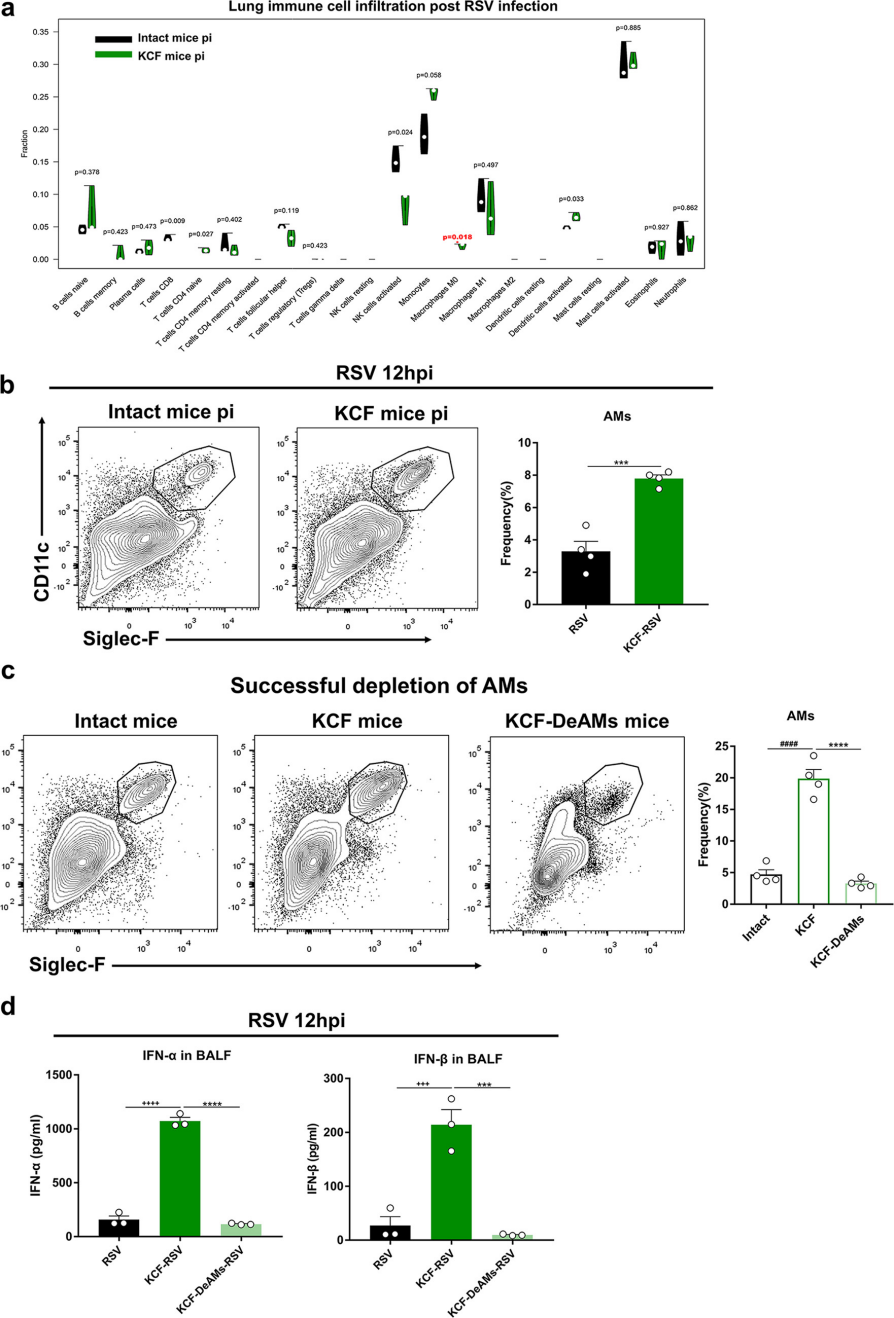
C-fiber degeneration regulates AMs to mediate resistance to RSV infection. (a) Violin diagram of proportions of 22 types of immune cells in lungs of KCF and intact mice post-RSV infection. (b) AM percentages in lung tissues of KCF and intact mice post-RSV infection, determined by flow cytometry. (c) AM percentages in lung tissues of intact mice, KCF mice, and KCF mice with clodronate-encapsulated liposomes. (d) IFN-α/β secretion as measured in BALF samples post-RSV infection in mice with or without AM deletion. *, *P* < 0.05, ***, *P* < 0.001, and ****, *P* < 0.0001, compared with intact mouse pi (postinfection) group or KCF-RSV group; ^+++^, *P* < 0.001, and ^++++^, *P* < 0.0001, compared with RSV group; ^####^, *P* < 0.0001, compared with intact group. Data are representative of 2 independent experiments. Error bars show SEM.

Here, we analyzed lung immune cell infiltration in KCF and intact mice at 12 h post-RSV infection. Among the 22 immune cells, macrophages were significantly increased in KCF mice post-RSV infection (Fig. 3a), as verified by flow cytometry (Fig. 3b). The gating strategy is shown in Fig. S3.

To determine whether C-fiber regulation of AMs mediates host resistance to RSV, we deleted AMs in KCF mice by using clodronate-encapsulated liposomes. The percentage of AMs in the lung decreased significantly after clodronate-encapsulated liposome treatment (Fig. 3c). IFN-α/β production as measured in BALF was greatly reduced at 12 h post-RSV infection in KCF mice with clodronate-encapsulated liposome treatment (Fig. 3d). These findings prove that C-fiber regulation of AMs has a direct role in IFN responses post-RSV infection.

### C-fiber degeneration upregulates VIP as measured in BALF.

**FIG 4:**
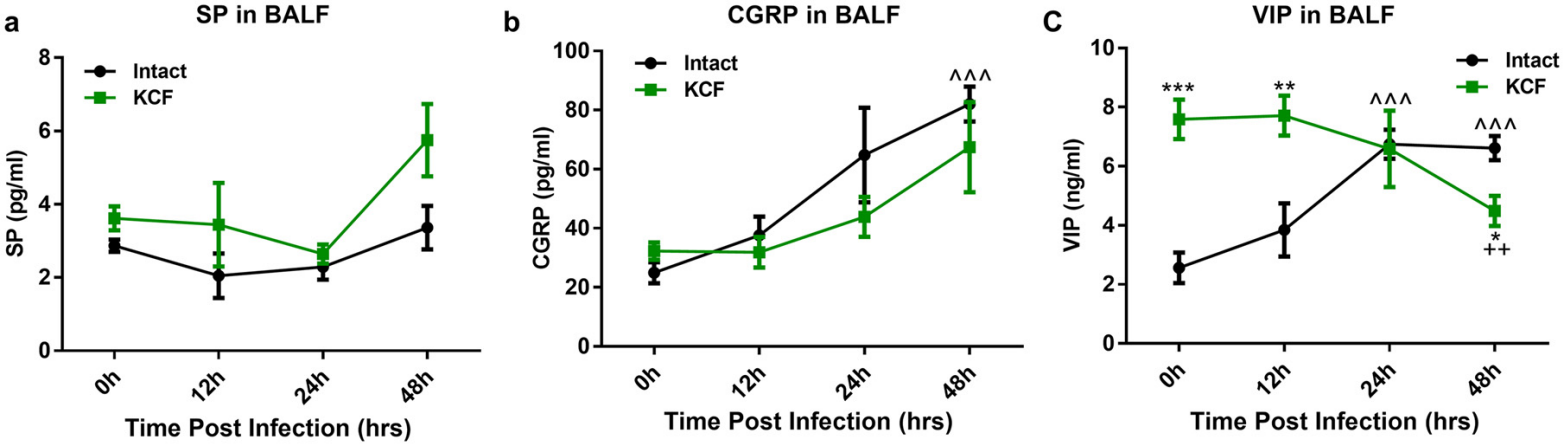
C-fiber degeneration upregulates VIP in BALF. SP, CGRP, and VIP in BALF were measured at 12, 24, and 48 h post-RSV infection in intact and KCF mice. C-fiber degeneration elevated secretion of VIP, even without infection. (a) SP levels. (b) CGRP levels. (c) VIP levels. *n* = 3 to 5/group. *, *P < *0.05, **, *P *< 0.01, and ***, *P *< 0.001, compared with intact RSV-infected mice at 48 hpi, intact RSV-infected mice at 12 hpi, and intact mice at 0 hpi; ^++^, *P < *0.01, compared with KCF mice at 0 hpi; and ^^^, *P < *0.001, compared with intact mice at 0 hpi. Data are representative of 3 independent experiments. Error bars show SEM.

C fibers can exert their biological effects by secreting/regulating levels of neuropeptides. The GSEA results shown in Fig. 1b also imply that antimicrobial peptides might play important roles in KCF mice post-RSV infection. Therefore, we quantified three important peptides, SP, CGRP, and VIP, in BALF using enzyme-linked immunosorbent assays (ELISAs). The results showed that SP levels remained unchanged at 12 to 48 h post-RSV infection in the intact and KCF mice (Fig. 4a), and the trends in the changes in CGRP levels were nearly parallel between the two groups (Fig. 4b). However, the basal levels of VIP (i.e., in uninfected mice), as well as the levels at 12 h postinfection, were significantly higher in KCF mice than in intact mice (Fig. 4c). These results show that C-fiber degeneration upregulated VIP content, which may mediate enhanced antiviral responses.

Next, we found that VIP mRNA expression was almost undetectable in the lung but was elevated in the intestine of KCF mice compared to the level in intact mice (Fig. S5a), which also had elevated VIP protein expression in the intestine (Fig. S5b). RNA-seq of the intestine of KCF and intact mice post-RSV infection revealed a significant positive correlation between intestinal VIP mRNA levels and lung IFN-α/β mRNA levels (Fig. S5c).

### VIP regulates AM and IFN-α/β responses post-RSV infection.

**FIG 5:**
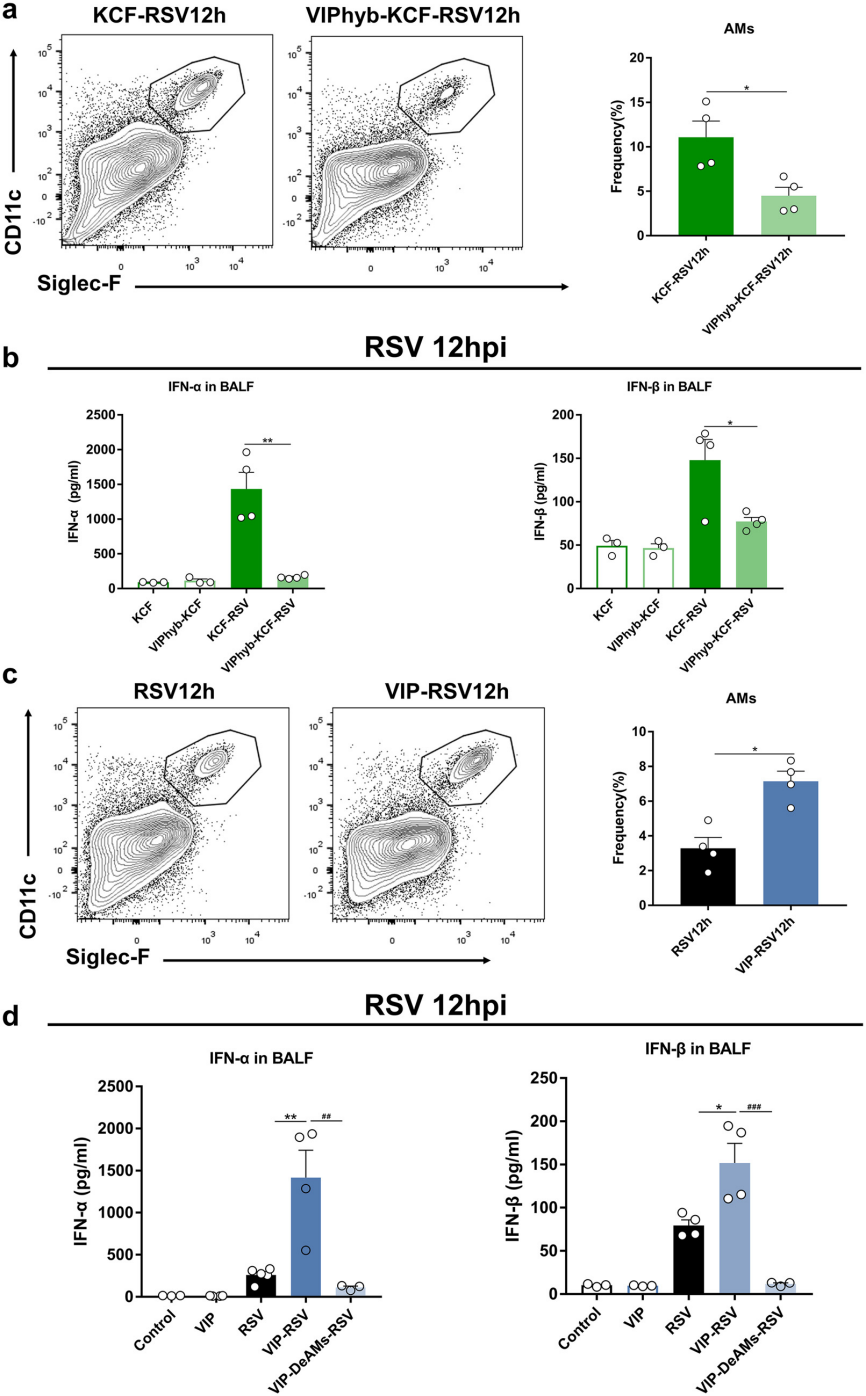
VIP regulates AM and IFN-α/β responses post-RSV infection. (a) AM percentages at 12 h postinfection in lung tissues of KCF mice with or without VIPhyb treatment. (b) IFN-α/β secretion at 12 h postinfection as measured in BALF samples from KCF mice with or without VIPhyb treatment. (c) AM percentages at 12 h postinfection in lung tissues of intact mice with or without VIP treatment. (d) IFN-α/β secretion at 12 h postinfection as measured in BALF samples from intact mice with or without VIP treatment. *, *P < *0.05, and **, *P *< 0.01, compared with RSV or KCF-RSV group; ^##^, *P < *0.01, and ^###^, *P *< 0.001, compared with VIP-RSV group. Data are representative of 2 independent experiments. Error bars show SEM.

We next determined whether VIP regulates AM and IFN-α/β responses at 12 h post-RSV infection. The results showed that the VIP receptor antagonist VIPhyb reduced the percentage of AMs and IFN-α/β production in KCF mice post-RSV infection (Fig. 5a and b). The results suggest that VIP mediates C-fiber regulation of AM and IFN-α/β responses post-RSV infection.

We next found that VIP increased the percentage of AMs and IFN-α/β production in intact mice post-RSV infection (Fig. 5c and d). To understand whether VIP regulation of AMs mediates VIP’s promotion of IFN-α/β production, we deleted AMs from VIP-treated mice with clodronate-encapsulated liposomes. Compared with the VIP-treated mice, the percentage of AMs in the lung decreased significantly after clodronate-encapsulated liposome treatment (Fig. S6). Treatment with VIP failed to restore IFN-α/β production as measured in BALF at 12 h post-RSV infection after deletion of AMs (Fig. 5d). The results confirmed that VIP regulation of AMs enhanced IFN-α/β production post-RSV infection.

### VIP mediates host defense against RSV infection.

**FIG 6:**
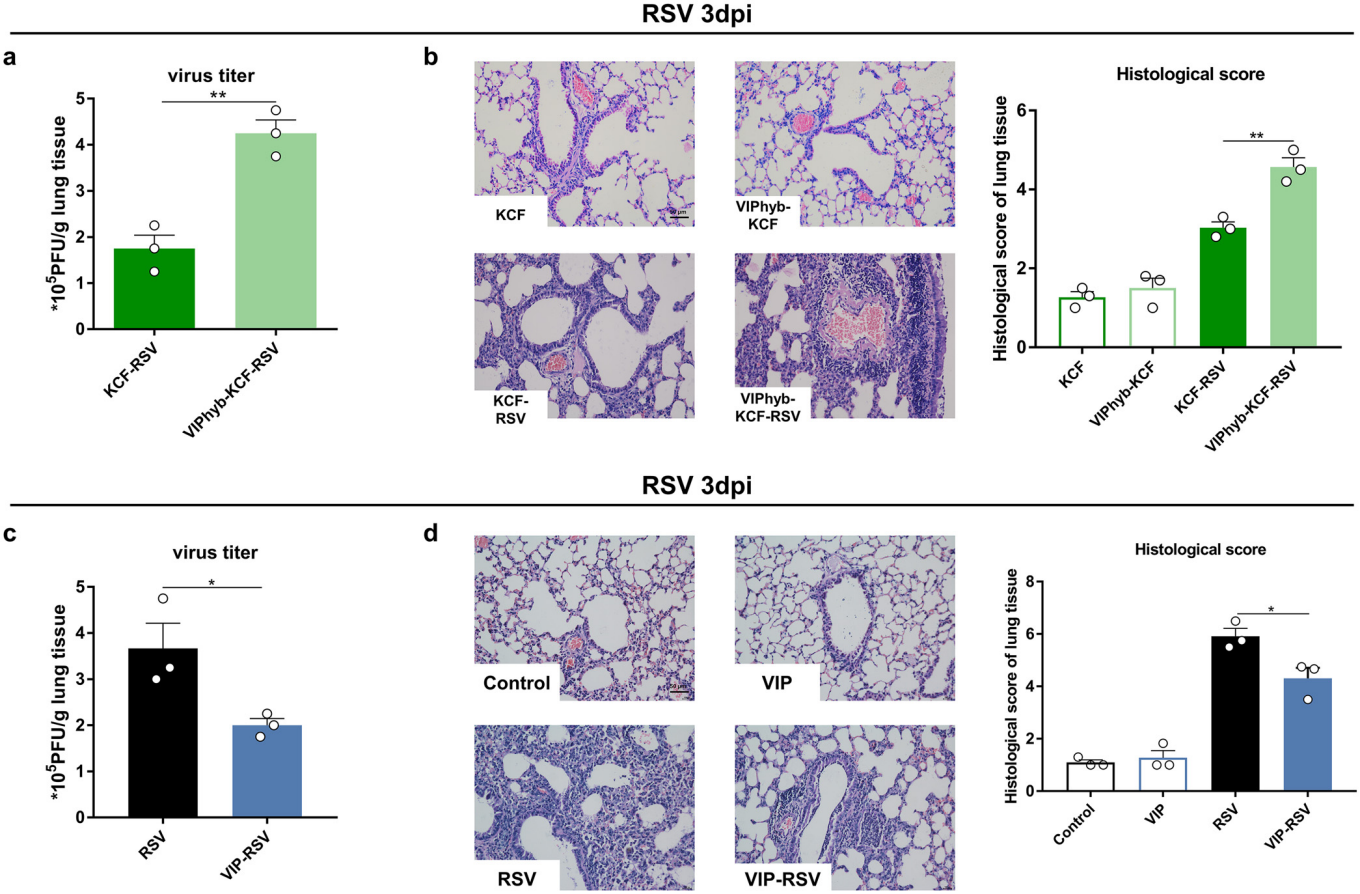
VIP protects against RSV infection. (a) RSV titers in lung tissues of KCF mice with or without VIPhyb treatment. (b) Representative lung histopathological images and scores of KCF mice with or without VIPhyb treatment. Scale bar = 50 μm. (c) RSV titers in lung tissues of intact mice with or without VIP treatment. (d) Representative lung histopathological images and scores of intact mice with or without VIP treatment. *, *P < *0.05, and **, *P *< 0.01, compared with RSV or KCF-RSV group. Data are representative of 2 independent experiments. Error bars show SEM.

To examine the functional role of VIP in host defense against RSV, we collected lung tissue samples to test RSV titers and lung inflammation at day 3 post-RSV infection. The results showed that VIPhyb promoted RSV replication and lung inflammation in KCF mice post-RSV infection (Fig. 6a and b). In contrast, VIP decreased RSV replication and lung inflammation in intact mice post-RSV infection (Fig. 6c and d). These results indicate that VIP protects against RSV infection.

## DISCUSSION

**FIG 7:**
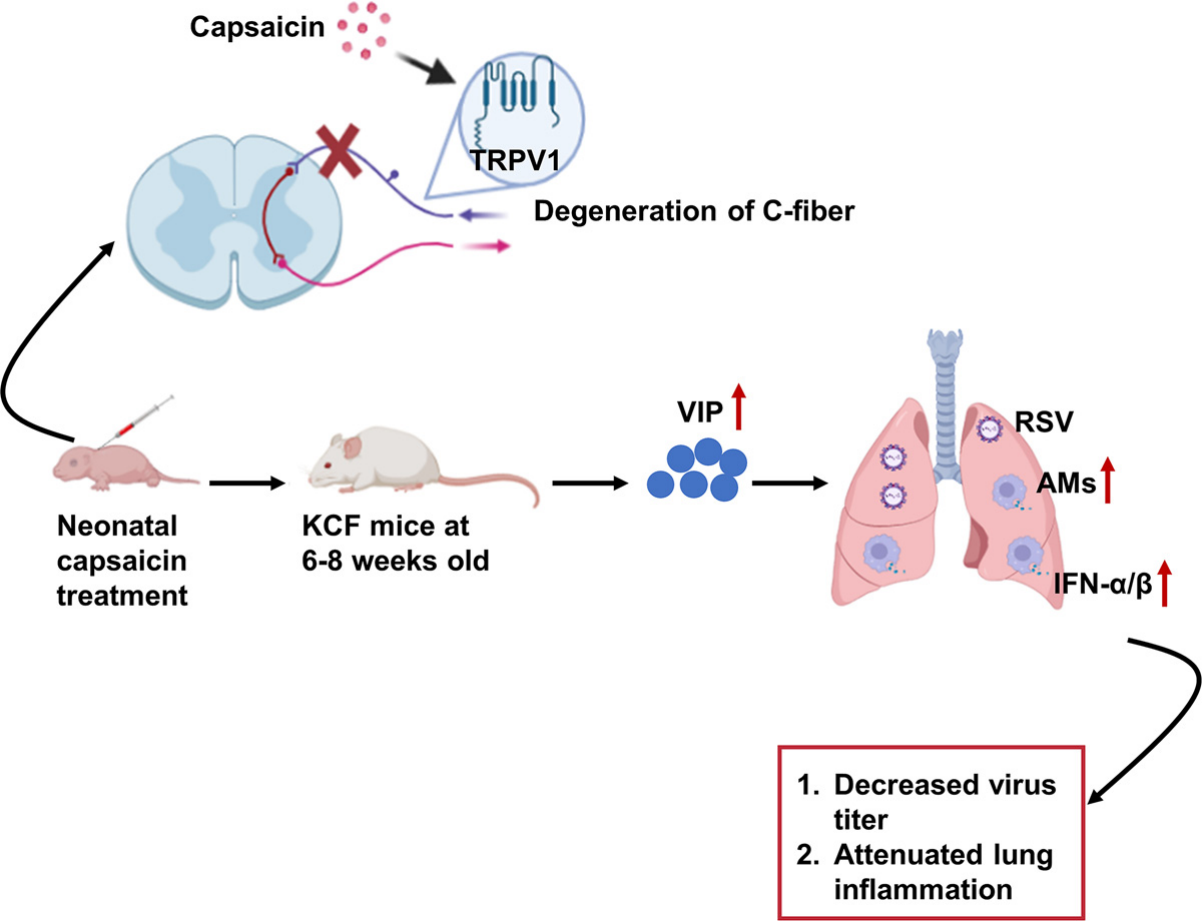
Overview of capsaicin treatment of neonatal BALB/c mice to cause degeneration of C fibers (KCF mice); KCF mice had elevated VIP as measured in BALF samples. The enhancement of AMs by the elevated VIP mediated the IFN-α/β response post-RSV infection in KCF mice, which contributed to decreased virus titers and attenuated inflammation.

A fundamental role of C fibers is to initiate host responses to viral infection. Here, we found that C-fiber degeneration enhanced host protective responses against RSV infection by regulating AM quantity to promote IFN-α/β secretion (Fig. 7). Furthermore, C fibers and AM and IFN-α/β responses were linked by VIP, which regulated both AM and IFN-α/β responses to RSV infection (Fig. 7). Thus, C-fiber and AM cross talk regulated lung defense against RSV infection.

The nervous and immune systems work in concert against pathogens and, thus, contribute to fighting infectious diseases (11). C fibers are the main afferent sensory nerves of the airways and lungs. In C fibers, TRPV1 is responsible for sensing various chemical and pathogenic stimuli in the environment. In *vivo*, RSV causes a persistent increase in the proinflammatory effects of sensory nerves, which is still present after the acute phase of the infection resolves (32). In *vitro*, RSV and rhinovirus can upregulate TRPV1 expression in sensory neurons (23, 33). We previously demonstrated that C-fiber degeneration inhibits RSV replication and decreases lung inflammation (26, 27). Here, we observed that C fibers almost disappeared in the lung and trachea of KCF BALB/c mice, consistent with previous findings in guinea pigs (28). Based on RNA-seq results, we further showed that degeneration of C fibers enhanced IFN-α/β responses post-RSV infection (Fig. 1b and c). TRPV1 blockade with a chemical antagonist was also effective at promoting IFN-α/β responses, suppressing viral replication, and alleviating lung inflammation post-RSV infection (Fig. 2a to c). Inhibition of TRPV1 decreases chikungunya virus replication, whereas activation of TRPV1 has the opposite effect (34). TRPV1 has also been widely studied in multiple inflammatory diseases. In chronic asthmatic mice, the administration of CPZ or TRPV1 small interfering RNA (siRNA) attenuates airway inflammation and hypersensitivity and reduces the levels of type 2 T helper cytokines and epithelial-cell-derived cytokines (35). Murine asthma models show upregulation in TRPV1 expression, which enhances the release of SP and CGRP and contributes to neurogenic inflammation, while CPZ treatment effectively reduces proinflammatory neuropeptides (36). Thus, our results and those of other studies reveal the important role of C fibers and TRPV1 in regulating host defense against viral infection, including RSV replication and inflammation.

AMs are essential for the host immune response against bacteria, fungi, and viruses and are the main source of IFN-α/β post-RSV infection (8, 37). Our results demonstrated that C-fiber degeneration upregulated the AM fraction post-RSV infection (Fig. 3a and b). After successful deletion of AMs in KCF mice, IFN-α/β were dramatically decreased post-RSV infection (Fig. 3c and d). These findings not only suggest that AMs are the main source of IFN-α/β production but also suggest that C-fiber and AM cross talk regulates host defense against viral infection.

C fibers usually regulate immune responses via CGRP and SP (13, 38, 39). In the present study, we found that SP and CGRP secretion levels were unchanged, but VIP was elevated in BALF samples from KCF mice compared with the level in intact mice (Fig. 4a to c). In pulmonary tissue, VIP is secreted by VIP-positive nerves and by several immune cells, such as macrophages, mast cells, granulocytes, and Th2 lymphocytes (40, 41). To clarify how C-fiber degeneration increases VIP secretion as measured in BALF, we performed RNA-seq of intestinal samples. We found that VIP mRNA and protein expression levels were elevated in the intestine, but VIP mRNA expression was almost undetectable in the lung (Fig. S5a and b). Additionally, VIP mRNA expression in the intestine was positively correlated with IFN-α/β expression in the lung post-RSV infection (Figure S5c). These results support the idea that VIP observed in BALF may originate from the intestine. Due to the common embryonic origin of the intestine and lung, the gastrointestinal and respiratory tracts exhibit structural and inherent immune response similarities (42). These similarities help the pulmonary-intestinal axis maintain homeostasis of the host’s internal environment during infection (43). Similar to our results, stimulation of the large intestine by gavage of mannitol or rhubarb significantly increases VIP both in the intestine and lung of chronic obstructive pulmonary disease (COPD) rats and asthmatic mice and reduces lung histopathological changes (44). To date, most studies have focused on the effects of gut flora on lung inflammation, lung injury, and disease progression (45). Notably, our study seemed to indicate that the pulmonary-intestinal axis may be interconnected through neuropeptides in addition to intestinal flora, but more experiments need to be done in the future to prove this speculation. As an important neuropeptide, VIP shows potent anti-inflammatory and immunomodulatory activities that affect both innate and adaptive immunity (40) and plays a critical role in the maintenance of the respiratory system homeostasis. Several animal and clinical trials suggest that VIP or VIP agonist treatment could be used for respiratory diseases, including acute lung injury, allergic asthma, and COPD (46–48). Our previous study showed that RSV-induced antiviral responses in KCF mice peak at 12 h postinfection, consistent with other research showing that IFN-α/β acts as a rapid-response cytokine upon RSV infection (49). In the current study, our results showed an increase in VIP secretion in the natural state and a high level at 12 h post-RSV infection, consistent with an enhanced antiviral response in KCF mice (Fig. 4c). We also showed that VIPhyb treatment suppressed AMs and IFN-α/β secretion and promoted viral replication and inflammation in KCF mice post-RSV infection (Fig. 5a and b and Fig. 6a and b). In contrast, exogenous VIP treatment promoted AMs and IFN-α/β secretion and decreased viral replication and inflammation in intact mice post-RSV infection (Fig. 5c and d and Fig. 6c and d). These results are consistent with previous reports. For example, VIP induces an antiviral state *in vitro* by stimulating IFN-α/β synthetase following vesicular stomatitis virus infection (50, 51). However, VIP has also been shown to promote cytomegalovirus (CMV) replication by inhibiting the Th1 antiviral response; moreover, pharmacological inhibition of VIP signaling or absence of VIP expression in VIP knockout (KO) mice has been shown to enhance antiviral immunity to CMV infection *in vivo* (52, 53). Collectively, these results suggest that the effects of VIP on type I IFN induction or viral replication can vary depending on the virus.

This study furthers our understanding of the role of the nervous system in host defense. Notably, we found that lung-innervating C fibers modulate several layers of lung physiology, including regulation of VIP, AMs, and IFN-α/β responses against RSV. Thus, VIP agonists and TRPV1 inhibitors may be potential therapeutic targets for the treatment of RSV infection.

## MATERIALS AND METHODS

### Ethics statement.

All experiments involving animals were carried out in accordance with the *Guide for the Care and Use of Laboratory Animals* (54) and were approved by the Institutional Animal Care and Committee (IACUC), which is authorized by the Association for Assessment and Accreditation of Laboratory Animal Care International of China and the Experimental Animal Committee of Chongqing Medical University [permits no. SCXK(Yu) 2012-0001 and SYXK(Yu) 2012-0001]. All surgeries were performed under anesthesia, and every effort was made to minimize pain.

### Virus preparation and infections.

The RSV-A2 strain (VR-1540) was obtained from the American Type Culture Collection (ATCC, Manassas, VA, USA). The strain was grown in HEp-2 cells in Dulbecco’s modified Eagle’s medium (DMEM) (C11995500BT; Gibco) containing 2% fetal bovine serum (FBS) (10100147; Gibco) and purified by density gradient (55). The virus titer was 1 × 10^8^ PFU/mL, as determined by serial dilution plaque assay (56). BALB/c mice were infected with 60 μL of RSV-A2 via intranasal (i.n.) inoculation under 2% to 5% isoflurane. Based on our previous study of lung tissue from RSV-infected mice, where antiviral IFN-α/β expression peaked at 12 h postinfection and virus replication peaked at day 3 postinfection, we chose the 12-h and 3-day time points for sampling (27).

### Animals.

Female BALB/c mice (6 to 8 weeks old) and perinatal BALB/c mice were purchased from the Animal Laboratory of Chongqing Medical University (China). Perinatal BALB/c mice were used to obtain neonatal mice for capsaicin (CAP) (catalog no. HY-10448; MedChemExpress) pretreatment. Mice were housed in individually filtered cages and provided with a diet of rodent chow and water. Mouse pups born by spontaneous vaginal delivery were housed with their mother and siblings (24 to 25°C and a 12 h/12 h light/dark cycle).

### Degeneration of C fibers.

Two days after birth, the mice were subcutaneously injected with CAP delivered in 10% Tween 80 and 10% ethanol in 0.9% NaCl, at a dose of 50 mg/kg of body weight (57). In detail, CAP was dissolved at a concentration of 4 mg/mL and was injected into neonatal mice in a volume of 12.5 mL/kg of body weight. A 2-day-old newborn BALB/c mouse was approximately 2 g in weight. The volume of CAP for subcutaneous (s.c.) injection for each neonatal mouse was about 25 μL. The females were exposed to multiple treatments at 6 to 8 weeks old. To confirm C-fiber degeneration after CAP pretreatment, we applied the ophthalmic test (58). Briefly, 20 μL of 0.01% (wt/vol) capsaicin was instilled into one eye, and the number of wiping motions that occurred in the subsequent 1-min period was counted. The females (6 to 8 weeks old) that wiped their eyes five times or fewer were used in the following experiments.

### CPZ and CAP treatments.

The TRPV1 receptor antagonist capsazepine (CPZ) (MedChemExpress, HY-15640) and CAP were each dissolved in a solution containing 0.9% NaCl, Tween 80, and ethanol at a ratio of 8:1:1. CPZ (dose of 15 mg/kg) and CAP (dose of 1 mg/kg) were administered by subcutaneous (s.c.) injection (59). Within 30 min, the mice were intranasally (i.n.) inoculated with RSV under 2% to 5% isoflurane anesthesia. Bronchoalveolar lavage fluid (BALF) samples were collected at 12 h post-RSV infection, and lung tissue samples at 3 days postinfection.

### VIP and VIPhyb treatments.

Both VIP and the VIP receptor antagonist VIPhyb were synthesized by GL Biochem Ltd. (Shanghai, China), with purities of >98%. All peptides were reconstituted with phosphate-buffered saline (PBS), stored at −80°C, and used within 1 week. C-fiber-degenerated (KCF) mice were treated intraperitoneally (i.p.) with VIPhyb at a dose of 10 μg (in 100 μL of PBS) (53). Intact mice were treated (i.p.) with VIP at a dose of 15 μg (in 150 μL of PBS) (60). All peptides were administered once daily for 5 days before RSV inoculation and continuously thereafter to the day before sacrifice. The synthetic sequences of VIPhyb and VIP are given in Table 1.

**TABLE 1:** Sequences of VIPhyb and VIP

Peptide	Sequence
VIPhyb	His-Ser-Asp-Ala-Val-Phe-Thr-Asp-Asn-Tyr-Thr-Arg-Leu-Arg-Lys-Gln-Met-Ala-Val-Lys-Lys-Tyr-Leu-Asn-Ser-Ile-Leu-Asn-NH2
VIP	Lys-Pro-Arg-Arg-Pro-Tyr-Thr-Asp-Asn-Tyr-Thr-Arg-Leu-Arg-Lys-Gln-Met-Ala-Val-Lys-Lys-Tyr-Leu-Asn-Ser-Ile-Leu-Asn-NH2

### Immunofluorescence.

The KCF and intact mice were first anesthetized by carbon dioxide and then euthanized by cervical dislocation. The trachea and lungs were removed and fixed in 4% paraformaldehyde for 24 h. After washing with PBS, the tissues were dehydrated in 15% sucrose (PBS dilution) for 30 min and then in 30% sucrose for 24 to 72 h (until they sank). The trachea and lung tissues were then embedded in optimal cutting temperature (OCT) compound and sectioned at a thickness of 12 μm on a cryostat (Leica CM1950). Prior to staining, the sections were incubated at room temperature successively in PBS containing 0.2% Triton X-100 for 30 min and PBS containing 0.3% hydrogen peroxide for 30 min, followed by avidin and biotin site blockade using PBS containing 5% bovine serum albumin (catalog no. 8020; Solarbio) for 1 h at room temperature. Sections were subsequently incubated with polyclonal anti-CGRP antibody (1:100) (PC205L; Millipore) overnight at 4°C in a wet box. After washing with PBS, the sections were incubated with fluorescein isothiocyanate (FITC)-labeled goat anti-rabbit IgG (1:200) (A0562; Beyotime) for 1 h at room temperature (avoiding light). The sections were then washed with PBS and immediately sealed with antifade mounting medium (P0126; Beyotime). The sections were imaged on a Leica epifluorescence microscope using FITC filter blocks.

### RNA-seq.

Twelve lung and 12 intestine (1 to 2 cm of colon near the ileocecal region) samples were randomly selected from intact, KCF, RSV12h (intact mice at 12 h post-RSV infection), and KCF-RSV12h (KCF mice at 12 h post-RSV infection) groups (three for each group and each organ). Total RNA was extracted using TRIzol reagent (catalog no. 15596018; Invitrogen) following the manufacturer’s procedures. Total RNA quantity and purity were analyzed using the Bioanalyzer 2100 and RNA 6000 Nano LabChip kit (Agilent, CA, USA), with an RNA integrity number (RIN) of >7.0. The poly(A) mRNA was isolated from purified total RNA with poly(T) oligonucleotide-attached magnetic beads (Invitrogen). After purification, the mRNA was fragmented into ~300-nucleotide-long oligonucleotides using divalent cations at an elevated temperature. The cleaved RNA fragments were then reverse transcribed using the dUTP method according to the protocols of the mRNA-seq sample preparation kit (Illumina, San Diego, USA) to create the final cDNA library, with an average insert size of 300 bp (±50 bp). Paired-end 2 × 150 bp (PE150) sequencing was performed on the Illumina NovaSeq 6000 platform by LC-Bio Bio-Tech Ltd. (Hangzhou, China), following the recommended protocols.

### RNA-seq data processing and GSEA.

Raw counts were transformed by vst conversion, and differentially expressed genes (DEGs) in the mouse groups were determined using the Bioconductor package DESeq2 (version 1.26.0) (61). To further investigate the functional alterations correlated with C-fiber degeneration post-RSV infection, we conducted gene set enrichment analysis (GSEA). The clusterProfiler package was used for GSEA based on gene expression profiles. Previously annotated gene sets Mm.c2.cp.reactome.v7.1.entrez.gmt and Mm.c2.cp.kegg.v7.1.entrez.gmt were chosen as the reference gene list. A default algorithm with 1,000 permutations was applied to calculate *P* values and enrichment scores. The gene set size filters were a minimum of 10, maximum of 1,000, *P* = 0.05, and pAdjustedMethod = BH. The cut-off criteria were set to a |normalized enrichment score (NES)| of >1 and a *P* value of <0.01.

### RNA isolation and real-time quantitative PCR (qRT-PCR).

Total RNA in lung tissues was extracted using an RNA extraction kit (TR205; Tianmo Biotech). Total RNA concentration and purity were assessed using a NanoDrop 2000, and 1 μg of RNA was used for first-strand cDNA synthesis using a PrimeScript RT kit (catalog no. RR047A; TaKaRa) according to the manufacturer’s instructions. The cDNA was amplified using a ChamQ universal SYBR qPCR master mix (catalog no. Q711-02; Vazyme) with the following primers: for *Ifna*, forward, 5′-AAGCCATCCTTGTGCTAAGAGA-3′, and reverse, 5′-AGCAAGTTGGTTGAGGAAGAGA-3′; for *Ifnb*, forward, 5′-AGCTCCAAGAAAGGACGAACA-3′, and reverse, 5′-GCCCTGTAGGTGAGGTTGAT-3′; and for *Gapdh* (encoding GAPDH [glyceraldehyde-3-phosphate dehydrogenase]), forward, 5′-CATCACTGCCACCCAGAAGACTG-3′, and reverse, 5′-ATGCCAGTGAGCTTCCCGTTCAG-3′. The PCR cycle conditions were 95°C for 2 min, then 40 cycles of 95°C for 5 s and 59°C for 30 s. Fold change values were obtained using the cycle threshold (2^−ΔΔ^*^CT^*) method with *Gapdh* calibration. The RSV nucleocapsid (N) gene copies were amplified using the following primers and probes: forward, 5′-AGATCAACTTCTGTCATCCAGCAA-3′; reverse, 5′-TTCTGCACATCATAATTAGGAGTATCAAT-3′; and probe, 5′-6-carboxyfluorescein (FAM)-CACCATCCAACGGAGCACAGGAGAT-Black Hole Quencher 1 (BHQ1)-3′. The PCR cycle conditions were 50°C for 2 min, 95°C for 10 min, then 40 cycles of 95°C for 15 s, and 60°C for 1 min. For absolute quantification, the RSV N gene copy number was calculated using a plasmid DNA standard curve.

### ELISA.

BALF was collected by flushing the lungs six times with 0.5 mL of PBS, with a recovery rate of >80%. One centimeter of colon near the ileocecal region was taken from intact and KCF mice. After removing the contents, intestines were weighed to add cold PBS (pH 7.0 to 7.2, precooled at 4°C, 1 mL/0.1 g) and then homogenized on ice using a glass homogenizer. The mixture was centrifuged at 5,000 × *g* for 10 min at 4°C, and the supernatant was collected and stored at −80°C for subsequent VIP determination. IFN-α (catalog no. BMS6027; eBioscience), IFN-β (catalog no. 439407; BioLegend), SP (item no. 583751; Cayman), CGRP (catalog no. E-EL-M0215c; Elabscience), and VIP (EIA-VIP; RayBiotech) were measured and calculated by enzyme-linked immunosorbent assay (ELISA) using commercial kits according to the manufacturers’ instructions.

### Lung tissue viral titer quantification.

Mice treated with VIP and VIPhyb were sacrificed at day 3 post-RSV inoculation. The lungs were removed and homogenized, and the supernatants were collected to determine lung viral titers using a plaque assay (56).

### Histopathological staining.

Lung tissues were fixed in 4% paraformaldehyde for 24 h, dehydrated, embedded in paraffin, and cut into 4-μm-thick sections. The sections were then stained with hematoxylin and eosin to assess airway inflammation. Inflammatory cell infiltration scores were evaluated in a double-blind manner by two independent investigators (62, 63).

### Immune cell infiltration in lungs of intact and KCF mice post-RSV infection based on RNA-seq.

The deconvolution algorithm contains gene expression reference values from a signature matrix of 547 genes in 22 types of immune cells (64). To evaluate the abundances of immune infiltrates in various organs, we uploaded the gene expression matrix data to CIBERSORT (https://cibersort.stanford.edu/), and the algorithm was run using the LM22 signature for 100 permutations. The percentage of each immune cell type in the samples was calculated and is displayed in a bar plot. The vioplot package (version 0.3.7) (65) was then used to draw violin diagrams to visualize differences in immune cell infiltration. The Wilcoxon test was used to analyze differences in immune cell fractions between the intact and KCF mice post-RSV infection.

### *In vivo* AM depletion.

For AM depletion, amounts of 100 μL of clodronate-encapsulated liposomes (CP-005-005; Liposoma) were given to uninfected KCF mice or VIP-treated mice (treated simultaneously with both clodronate-encapsulated liposomes and VIP) by i.n. instillation every other day (66). On the fifth day, some mice were randomly selected to be sacrificed, and their lungs were collected for flow cytometry analysis to assess AM depletion. Other mice were infected intranasally with RSV.

### Single-cell-suspension preparation and flow cytometry.

Lungs were digested for 30 min at 37°C in Roswell Park Memorial Institute (RPMI) 1640 culture medium (Gibco) containing 2 mg/mL collagenase IV (product no. C0130; Sigma) and 30 μg/mL DNase I (10104159001; Roche). The samples were then passed through a 70-μm cell strainer to obtain a single-cell suspension. The cells were blocked with rat serum for 30 min and then stained with fixable viability dye eFluor 660 (catalog no. 65-0864-14; Invitrogen), phycoerythrin (PE)-Cy7-conjugated anti-CD45 antibody (17-0451-82; eBioscience), FITC-conjugated anti-CD11c antibody (11-0114-82; eBioscience), and peridinin chlorophyll protein (PerCP)-Cy5.5-conjugated anti-Siglec-F (sialic acid-binding Ig-like lectin F) antibody (155526; BioLegend) in the dark for 1 h. Samples were analyzed on a BD FACSCanto plus equipped with 50-mW 405-nm, 50-mW 488-nm, and 20-mW 633-nm lasers and an ND1.0 filter in front of the forward scatter (FSC) photodiode. Flow cytometry results were analyzed using FlowJo software (version 10, TreeStar, USA), and CD45^+^ Siglec-F^hi^ CD11c^hi^ cells were identified as AMs.

### Statistical analysis.

Data were analyzed using GraphPad Prism version 7.0 (GraphPad Software, Inc., San Diego, CA, USA). All results were expressed as mean values ± standard errors of the means (SEM). For comparisons of two groups, the statistical significance was estimated using the unpaired two-tailed Student’s *t* test. Multiple group comparisons were performed using analysis of variance (ANOVA). A *P* value of <0.05 was considered statistically significant.

### Data availability.

The RNA sequencing data have been deposited in the GEO database and are accessible through GEO Series accession numbers GSE197868 and GSE215099.
