# Supplementary material for: C-Fiber Degeneration Enhances Alveolar Macrophage-Mediated IFN-α/β Response to Respiratory Syncytial Virus
Source: Microbiol Spectr. 2022 Nov 9;10(6):e02410-22. doi: 10.1128/spectrum.02410-22 (PMC9769737; doi:10.1128/spectrum.02410-22)
Supplement: Supplemental file 1 — Fig. S1 to S6. Download spectrum.02410-22-s0001.pdf, PDF file, 0.9 MB [file spectrum.02410-22-s0001.pdf]

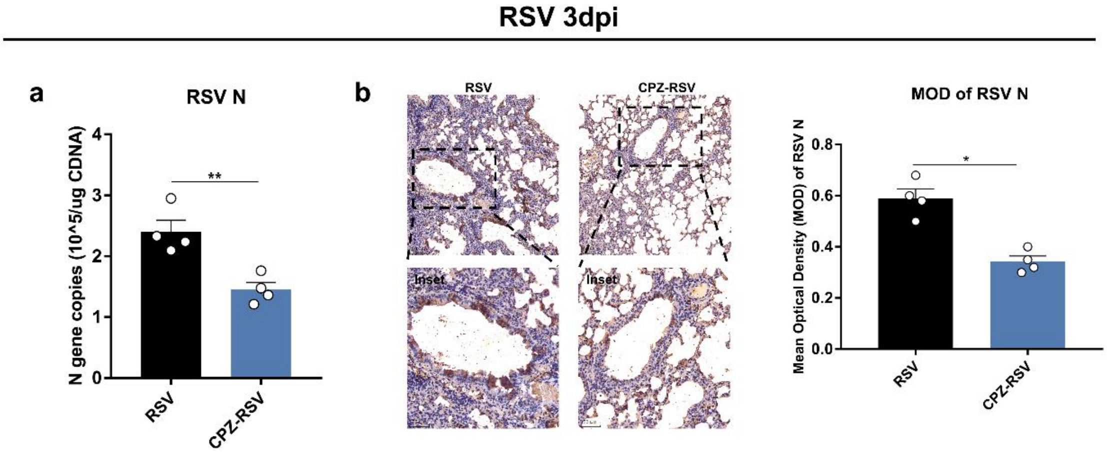

Figure S1. C-fibers facilitate RSV replication. (a) RSV N gene copies by qRT-PCR. (b) Representative histochemical images and mean optical density (MOD) of RSV N protein. *, **, $P < 0.05$, 0.01, compared with RSV group. Data are representative of 2 independent experiments.

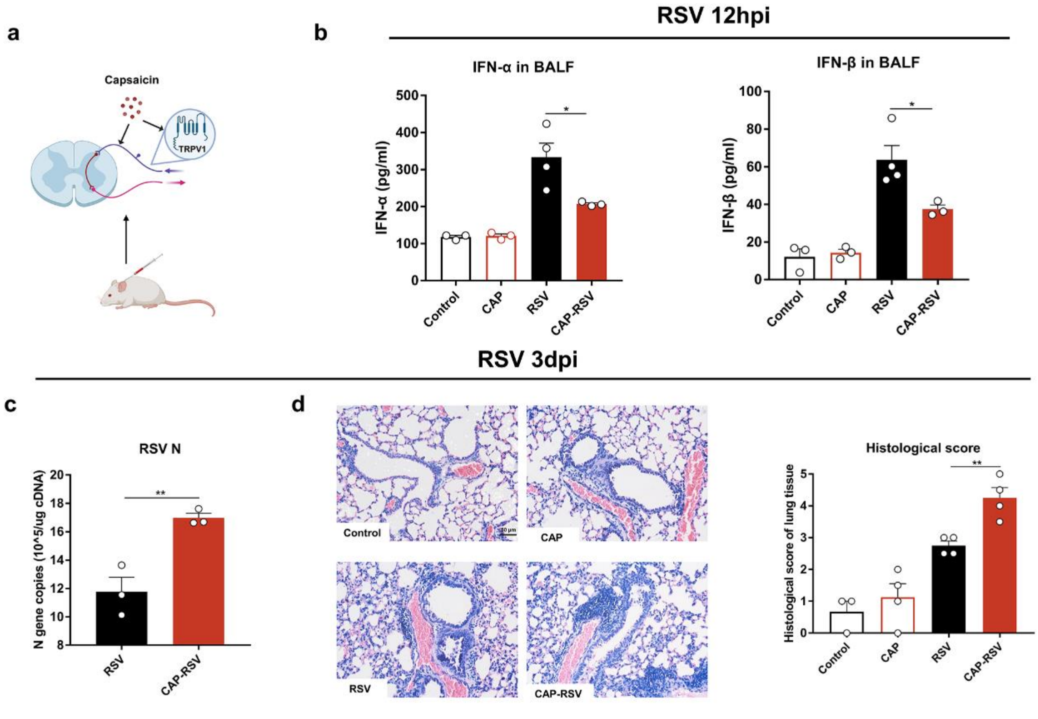

Figure S2. Activation of TRPV1 impairs host defense against RSV infection. (a)

TRPV1 agonist CAP treatment in intact mice. (b) IFN-α/β secretion in BALF with or without CAP treatment. (c) RSV titers in lung tissue with or without CAP treatment. (d) Representative lung histopathological images and scores with or without CAP treatment. * and **, compared with RSV group, $P < 0.05$ and < 0.01, respectively. Data are representative of 2 independent experiments.

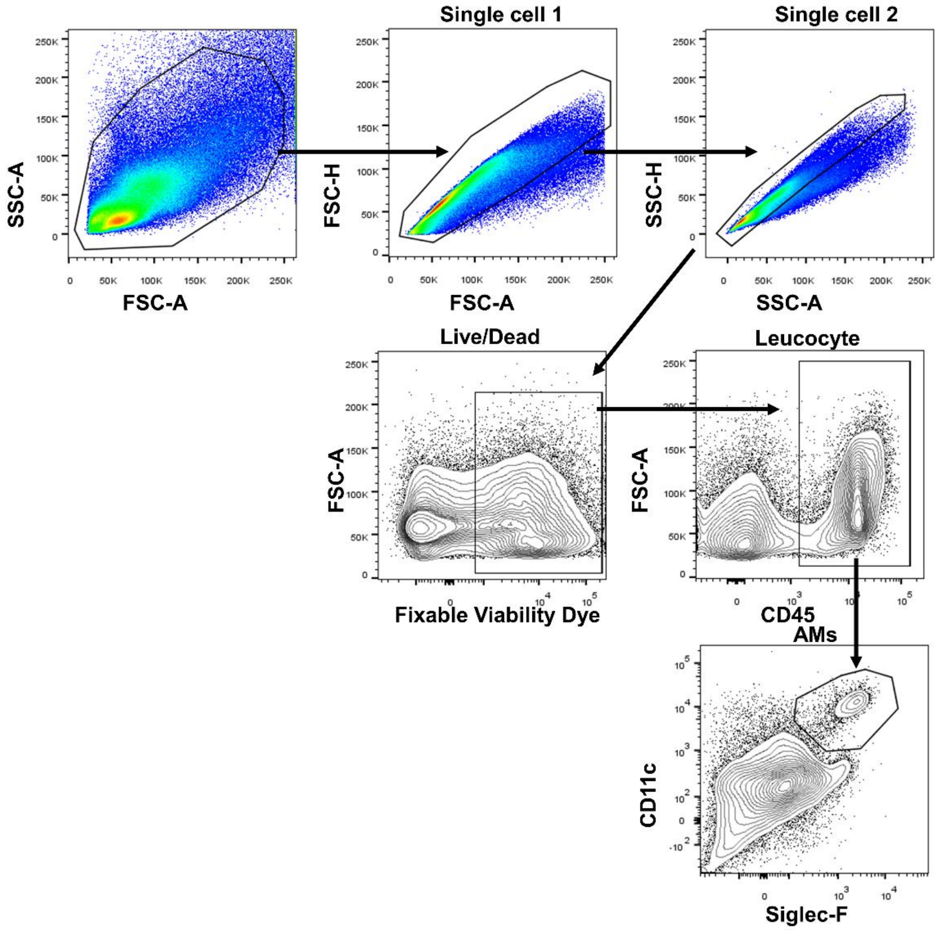

Figure S3. Gating strategy to identify AMs. Lung cells were obtained by collagenase IV and DNase I digestion and stained with indicated cell-surface molecules.

Represented gates were used to identify AMs. Cell debris were removed by FSC SSC-based and singlet gating. Dead cells were excluded by fixable viability dye staining. $CD45^{+}SiglecF^{hi}CD11c^{hi}$ cells were regarded as AMs.

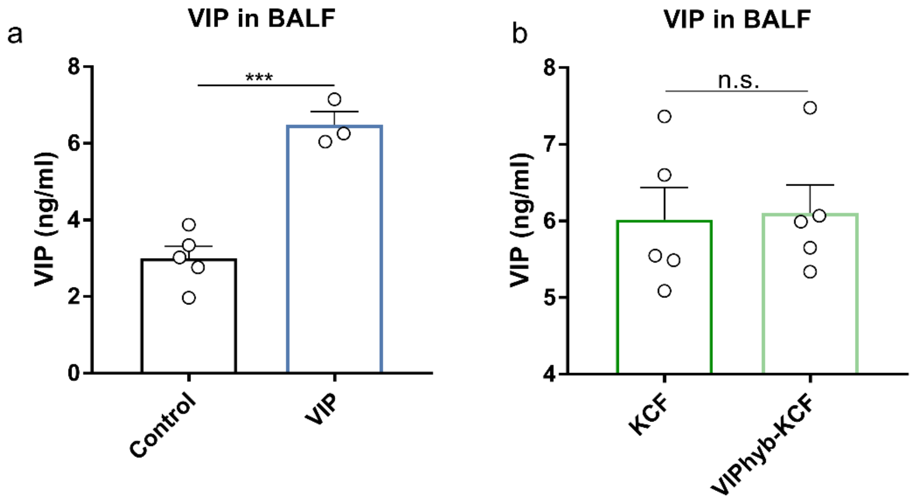

Figure S4. VIP injection increased VIP concentration in BALF. (a) VIP concentration in BALF after VIP injection for five days. (b) VIP concentration in BALF after VIPhyb injection for five days. ***, $P < 0.001$, compared with control group. n.s., no significance. Data are representative of 2 independent experiments.

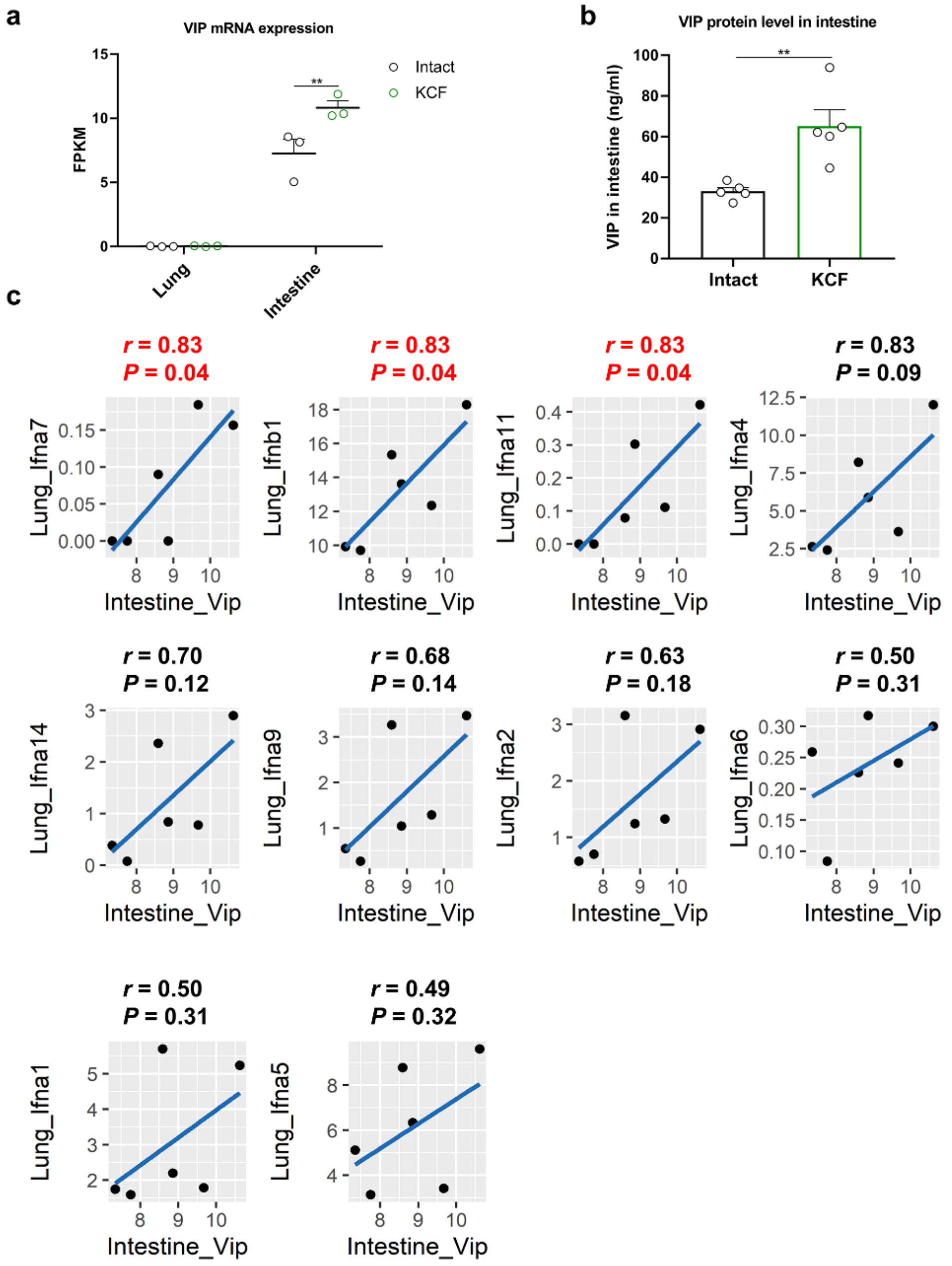

Figure S5. Correlation of VIP expression in intestine with IFN-α/β expression in lung post-RSV infection. (a) VIP mRNA expression in lung and intestine between KCF and intact mice based on RNA-seq. (b) VIP protein level in intestine between KCF and intact mice with ELISA. (c) Correlation of VIP mRNA expression in intestine with IFN-α/β mRNA expression in lung of KCF and intact mice at 12 h post-RSV infection. **, $P < 0.01$, compared with intact mice. Data are representative of 2 independent

experiments.

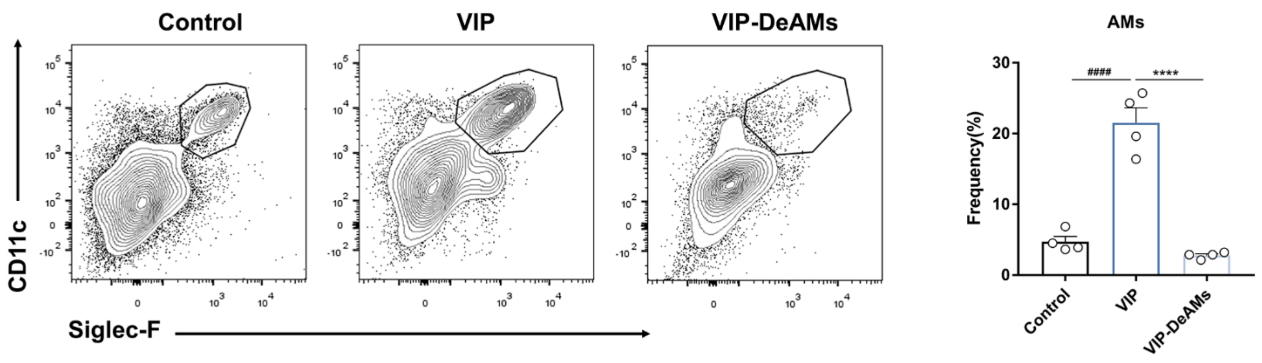

Figure S6. AM percentage in lung tissue of VIP-treated mice with or without clodronate-encapsulated liposomes. Clodronate-encapsulated liposomes significantly reduced AMs in VIP-treated mice. ****, ####, *P* < 0.0001, compared with VIP and control group respectively. Data are representative of 2 independent experiments.
